# OCCUPATIONAL COGNITIVE DEMANDS AND RISK OF MILD COGNITIVE IMPAIRMENT AND DEMENTIA: THE HUNT STUDY

**DOI:** 10.1093/geroni/igad104.3502

**Published:** 2023-12-21

**Authors:** Bjorn Heine Strand, Trine Holt Edwin, Ekaterina Zotcheva, Asta Håberg, Bernt Bratsberg, Teferi Mekonnen, Yaakov Stern, Vegard Skirbekk

**Affiliations:** Norwegian Institute of Public Health, Oslo, Oslo, Norway; Oslo University Hospital, Oslo, Oslo, Norway; Norwegian National Centre for Ageing and Health, Tønsberg, Vestfold, Norway; Norwegian University of Science and Technology, Trondheim, Sor-Trondelag, Norway; Ragnar Frisch Center for Economic Research, Oslo, Oslo, Norway; Norwegian Institute of Public Health, Oslo, Oslo, Norway; Columbia University, New York City, New York, United States; Norwegian Institute of Public Health, Oslo, Oslo, Norway

## Abstract

The cognitive reserve hypothesis posits that cognitively stimulating work may delay onset of mild cognitive impairment (MCI) and dementia. However, the impact of work histories across midlife on the risk of these conditions is unclear. We evaluated trajectories of work-related cognitive demands from ages 33-65y and their association with later clinical MCI/dementia. The HUNT4 70+ Study was used (N= 7003 participants, 49.8% women, 2017-19). Group-based-trajectory-modeling was used to identify trajectories of work-related cognitive demands, measured by routine task intensity (RTI) index (Lower RTI indicates less routine-oriented work) from online Occupational Information Network (O*NET). Multinomial regression was used to estimate the relative risk ratios (RRR) of MCI/dementia after adjusting for covariates age, sex and education, income, hypertension, obesity, diabetes, psychiatric impairment, hearing impairment, feeling of loneliness, smoking status, and physical inactivity. Four trajectories were identified; low RTI (20.4%), intermediate-low RTI (22.7%), intermediate-high RTI (36.9%), and high RTI (20.1%). Participants in the high RTI group had higher risk of MCI (RRR 1.74, 95% CI: 1.41,2.14) and dementia (RRR 1.37, 95% CI: 1.01, 1.86) after adjusting for age, sex, and education compared to participants in the low RTI group. When accounting for potential mediators, the increased risk remained for MCI but was no longer significant for dementia, indicating these mediators may account for part of the observed relationship. Our results highlight the importance of cognitive stimulation in the workplace for maintaining cognitive function. This should be taken into account, together with other known risk factors to help prevent cognitive decline in older adults.

